# Supplementary material for: Less Animal-Based Food, Better Weight Status: Associations of the Restriction of Animal-Based Product Intake with Body-Mass-Index, Depressive Symptoms and Personality in the General Population
Source: Nutrients. 2020 May 20;12(5):1492. doi: 10.3390/nu12051492 (PMC7284911; doi:10.3390/nu12051492)
Supplement: Supplementary file 1 [file nutrients-12-01492-s001.pdf]

Supplementary Material

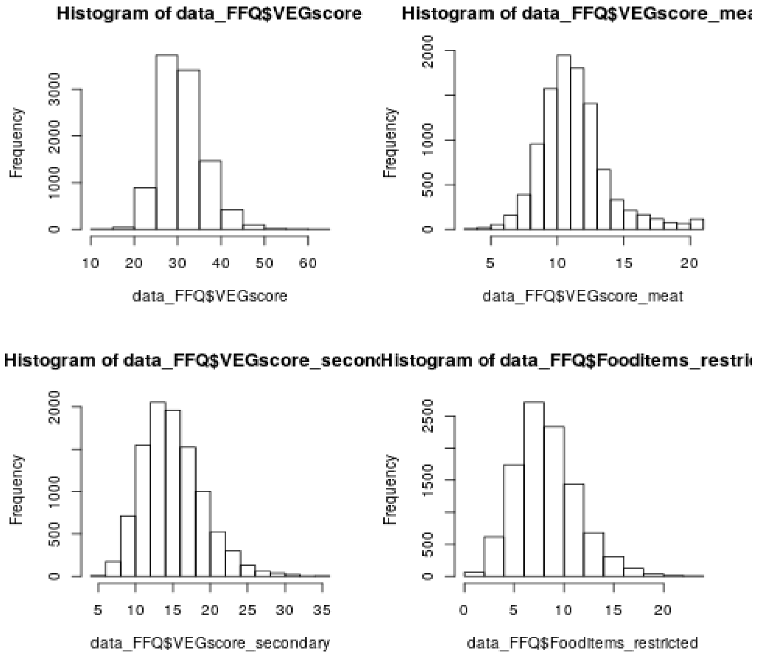

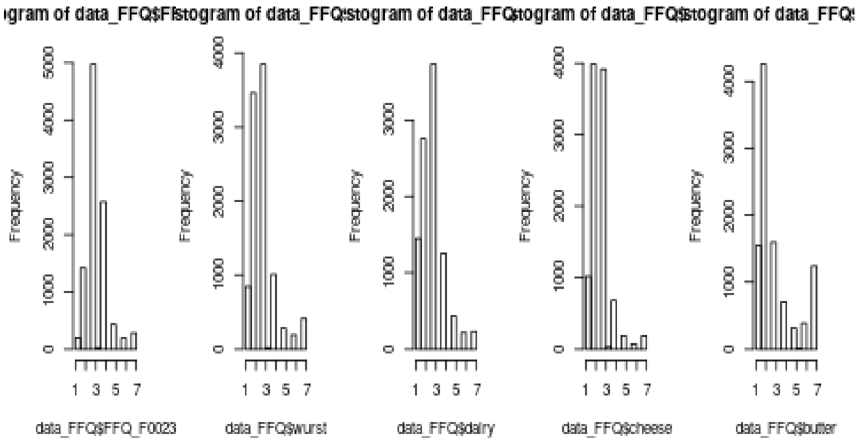

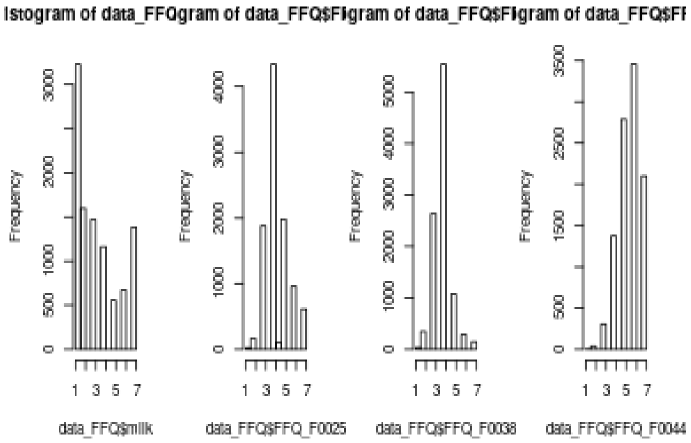

Figure S1. Frequency distribution of the dietary scores. A) animal DRS B) primary animal DRS C) secondary animal DRS and D) overall DRS. All scores are normally distributed (skewness >0.5 and <1). E) Frequency distributions of 9 items used in animal DRS.

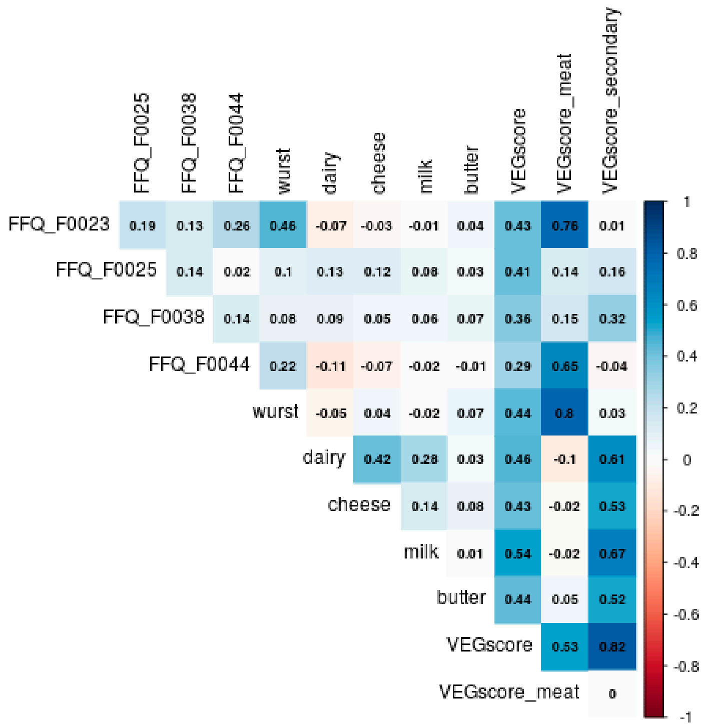

Figure S2. Correlation plot of nine items included in animal DRS.

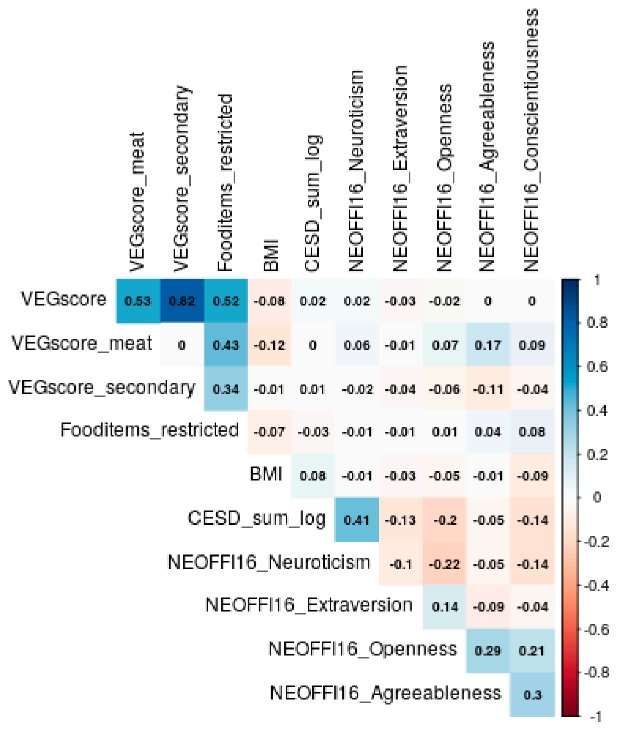

Figure S3. Correlation plot of all measures of interest including dietary patterns, BMI, CES-D and personality traits.

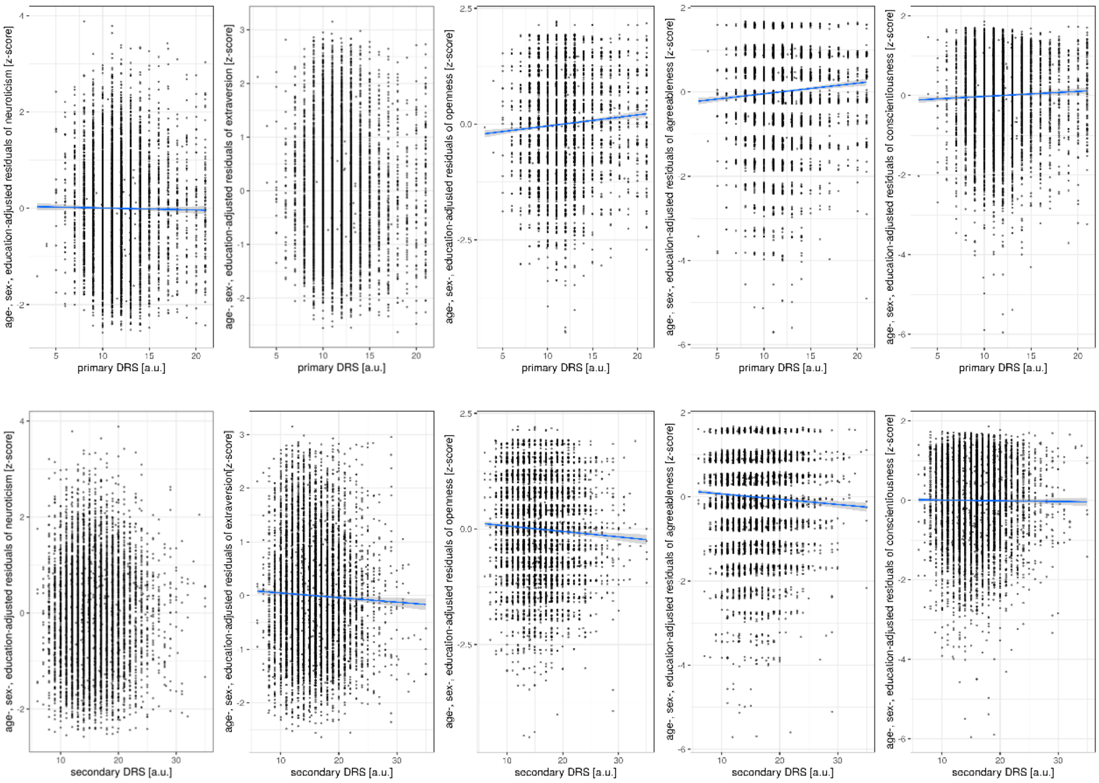

Figure S4. Associations frequency of animal-based products and personality traits (top row: primary DRS; bottom row: secondary DRS).

Table S1. Summary of computed dietary restriction scores.

		**animal DRS (9 - 63)**	**primary animal DRS (3 - 21)**	**secondary animal DRS (5 - 35)**	**overall DRS (0 - 33)**
**Sample 1 (n=8943)**	**mean**	31.5 (14-63)	11.7 (3-21)	15.5 (6-35)	8.7 (0-24)
	**SD**	5.1	2.6	4.0	3.1

Figure 1: Flowchart of sample selection for sample 1 and sample 2. 7
Figure 2: Concept of dietary restriction score (DRS) based on the frequency of consumption of animal-based products over the last 12 months based on 9 items from the FFQ. 9
Figure 3: Association between BMI and demographic and lifestyle factors A) animal DRS B) age, residuals plotted according to regression model 1 (sample 1 n = 8943). 10
Figure 4: Association between animal DRS and extraversion, residuals plotted according to regression model 2 (sample 1 n = 8943). 11
Figure 5: Significant association between personality traits and depressive symptoms in sample 2 (n = 7906) corrected for age, sex, education, animal DRS and the respective four other subscales of personality for neuroticism, extraversion, agreeableness, conscientiousness and BMI. 12
Figure 6: Restrictive animal-based product intake associated with lower BMI. 13
Figure 7: A) Positive association between decreasing frequency of animal-based products and number of excluded food groups. Negative association between overall dietary restriction score and B) BMI and C) CES-D. 14

Table 1: Demographic characteristics for sample 1 and sample 2. 22
Table 2: Personality traits according to the five factor personality questionnaire NEO-FFI (16 items) for sample 2 (n=7,906). 23
Table 3: Multiple regression analyses predicting BMI as function of age, sex, education and frequency of animal-based products (n = 8943). 24
Table 4: MANCOVA analysis of animal DRS, age, sex, education on personality (n = 7906). 25
Table 5: Multiple regression analyses predicting CES-D as a function of age, sex, education animal DRS (sample 1, n=8493) and additionally personality traits (sample 2, n = 7906) and BMI. 27
Table 6: Multiple regression analyses predicting BMI as a function of age, sex, education and restriction of different dietary items (sample 1, n=8493). 29
Table 7: Multiple regression analyses predicting CES-D as a function of age, sex, education and primary and secondary dietary restriction score (sample 1 n = 8943, sample 2 n = 7906). 30
Table 8: MANCOVA analysis of dietary restriction, age, sex, education on personality (n = 7906). 32
Table 9: Multiple regression analyses predicting CES-D as a function of age, sex, education and dietary restriction score (sample 1 n = 8943, sample 2 n = 7906). 33

Suppl. Figure 1: Frequency distribution of the dietary scores. 35
Suppl. Figure 2: Correlation plot of nine items included in animal DRS. 36
Suppl. Figure 3: Correlation plot of all measures of interest including dietary patterns, BMI, CES-D and personality traits. 36
Suppl. Figure 4: Associations frequency of animal-based products and personality traits (top row: primary DRS; bottom row: secondary DRS). 37

Suppl. Table 1: Summary of computed dietary restriction scores. 35
